# Efficiency and Optimization of Iodinated Contrast Agents in Cerebral Aneurysm CT Angiography: A Systematic Review

**DOI:** 10.3390/diagnostics16162666

**Published:** 2026-08-21

**Authors:** Raluca-Maria Lezeu, Razvan Chirla, Simona Daniela Cavalu

**Affiliations:** 1 Doctoral School of Biomedical Sciences, Faculty of Medicine and Pharmacy, University of Oradea, P-ta 1 Decembrie 10, 410087 Oradea, Romania; lezeu.ralucamaria@student.uoradea.ro; 2 Department of Preclinical Sciences, Faculty of Medicine and Pharmacy, University of Oradea, P-ta 1 Decembrie 10, 410087 Oradea, Romania

**Keywords:** cerebral CT angiography, intracranial aneurysm, iodinated contrast agent, low-dose protocol, low tube voltage, contrast volume reduction, ALADA principle

## Abstract

**Background/Objectives**: The demand for reducing iodinated contrast agent (ICA) dose in cerebral CT angiography (CTA) for aneurysm detection has increased due to concerns over patient safety and image quality. The aim of this study is to systematically review the diagnostic accuracy, image quality, and dose efficiency of optimized ICA protocols in CTA for intracranial aneurysm detection. We also aimed to distinguish between direct diagnostic studies reporting aneurysm sensitivity/specificity and indirect technical studies, emphasizing patient-specific tailoring using cardiac output and lean body weight. **Methods**: Following PRISMA 2020 guidelines, PubMed, Scopus, and Web of Science were searched up to 2 June 2026. Two reviewers independently screened studies, extracted data, and assessed risk of bias using QUADAS-2 and the NIH tool. Due to clinical and technical heterogeneity, a narrative synthesis was performed. **Results**: Eleven studies comprising 716 patients were included. Low tube voltage (70–80 kVp) combined with reduced iodine load (8–30 mL, 3.2–9 gI) maintained diagnostic accuracy for aneurysms >3 mm compared to standard protocols. Iterative reconstruction and deep learning improved contrast-to-noise ratio by 23–45% at reduced dose. Technical studies showed feasibility of ultra-low-volume protocols but lacked aneurysm-specific DSA validation. **Conclusions**: Reduced-contrast and low-kVp CTA protocols may preserve diagnostic performance for intracranial aneurysm detection while lowering iodine exposure. However, current evidence is limited by small sample sizes and heterogeneous protocols. Prospective, aneurysm-specific validation is required before clinical implementation.

## 1. Introduction

Intracranial aneurysms represent a significant global health burden. The rupture of these vascular anomalies leads to subarachnoid hemorrhage (SAH), a catastrophic event associated with high mortality and long-term neurological morbidity [1,2]. Consequently, rapid and accurate detection, as well as precise morphological characterization, are paramount for surgical planning and endovascular intervention.

Even though digital subtraction angiography (DSA) is the gold standard for diagnosing intracranial aneurysms due to its superior spatial resolution and dynamic flow assessment, it is considered invasive, uses a relatively high radiation dose, and causes complications [3,4]. Risks of thromboembolic complications, hemorrhage at the puncture site, and nerve injury were reported [5].

Multidetector-row computed tomography angiography (CTA) could be a promising alternative, although radiation exposure and contrast-associated acute kidney injury, especially in patients with renal insufficiency, hypovolemia, and diabetes mellitus, and in older patients [6], are still among the drawbacks [7]. Modern CT technology offers rapid acquisition and high diagnostic performance, making it the frontline modality in both emergency and elective settings. The diagnostic “efficiency” of CTA is fundamentally dependent on the intravascular opacification achieved by iodinated contrast agents (ICA). Achieving an optimal signal-to-noise ratio requires a delicate balance between contrast physicochemical properties, such as iodine concentration and viscosity, and injection dynamics, including flow rate and bolus tracking. However, the administration of ICA is not without risk, particularly regarding contrast-induced nephropathy and, more rarely, neurotoxicity [8,9].

Research highlights that higher iodine concentrations (e.g., 370–400 mgI/mL) provide superior attenuation but come with higher viscosity, which can limit the maximum flow rate feasible through standard intravenous cannulas. Balancing these factors with bolus tracking (triggering the scan at a specific Hounsfield Unit threshold) is essential to capture the peak arterial phase [10].

Cerebral CTA is the first-line non-invasive modality for detection and follow-up of intracranial aneurysms. The optimization of ICA administration aims to achieve diagnostic arterial enhancement while adhering to the ALADA “As Low As Diagnostically Acceptable” principle. Strategies include reduced contrast volume, low tube voltage, iterative reconstruction, spectral imaging, and patient-specific dosing. However, the interaction of these parameters and their impact on aneurysm detection remains unclear [11,12].

The main goal of this systematic review was to synthesize and evaluate the information regarding the efficiency and diagnostic accuracy of optimized iodinated contrast protocols in CTA for intracranial aneurysm detection. We also aimed to distinguish between direct diagnostic studies reporting aneurysm sensitivity/specificity and indirect technical studies, emphasizing patient-specific tailoring using cardiac output and lean body weight. This aneurysm-specific focus addresses a gap in current guidelines and provides a practical framework for ALADA-guided protocols in neurovascular imaging.

## 2. Research Methodology

This systematic review was conducted and reported in accordance with the PRISMA 2020 statement [13].

### 2.1. Eligibility Criteria

Studies were included if they met the following criteria based on PICOS:

• Population: Adult patients ≥18 years undergoing CTA for evaluation of suspected or known intracranial aneurysms.
• Intervention/Index test: CTA protocols using optimized iodinated contrast administration. This included reduced contrast volume, low tube voltage ≤ 80 kVp, iterative reconstruction, spectral CT, or patient-specific dosing strategies.
• Comparator: Standard CTA protocol or a different optimized protocol within the same study.
• Outcomes: At least one of the following: diagnostic accuracy for aneurysm detection using DSA as the reference standard; quantitative image quality metrics (HU, SNR, CNR); contrast volume; and radiation dose.
• Study design: Original clinical studies in humans. Case reports, reviews, editorials, conference abstracts, and animal studies were excluded. Studies evaluating coronary CTA, pulmonary embolism CTA, abdominopelvic CTA, or cone-beam CT were excluded from the primary analysis but discussed separately as indirect technical evidence in Section 4.5.

### 2.2. Information Sources and Search Strategy

PubMed, Scopus, and Web of Science were searched from inception to 2 June 2026 with no language filter other than English. The search combined terms related to “intracranial aneurysm”, “CT angiography”, and “iodinated contrast” using Boolean operators.

No filter for time-interval limitation was selected. The following search algorithm was applied in the PubMed database: (“Intracranial Aneurysm”[MeSH] OR “Cerebral Aneurysm”) AND (“Computed Tomography Angiography”[MeSH] OR “CTA”) AND (“Contrast Media”[MeSH] OR “Iodine” OR “Low-dose protocol”) AND (“Sensitivity and Specificity”[MeSH] OR “Diagnostic accuracy” OR “Efficiency”), with 10 records being identified. The search string applied for Web of Science was: TS = ((“intracranial aneurysm*” OR “cerebral aneurysm*” OR “brain aneurysm*”) AND (“CT angiography” OR “CTA” OR “computed tomograph* angiograph*”) AND (“iodinated contrast*” OR “contrast agent*” OR “contrast media” OR “iohexol” OR “iodixanol” OR “iopromide” OR “iopamidol”) AND (“efficiency*” OR “efficacy” OR “diagnostic accuracy” OR “sensitivity” OR “specificity” OR “image quality” OR “opacification”)), which resulted in 30 records. The search string applied for Scopus was: TITLE-ABS-KEY((“intracranial aneurysm*” OR “cerebral aneurysm*” OR “brain aneurysm*”) AND (“CT angiography” OR “CTA” OR “computed tomograph* angiograph*”) AND (“iodinated contrast*” OR “contrast agent*” OR “contrast media” OR “iodine” OR iohexol OR iodixanol OR iopromide OR iopamidol) AND (“low dose” OR “low tube voltage” OR “reduced contrast” OR “image quality” OR “diagnostic accuracy” OR “opacification” OR efficiency)), which resulted in 166 records. The final PRISMA Flow Diagram for this systematic review started with 206 identified records (30 from Web of Science, 10 from PubMed and 166 from Scopus) and tracked their selection through de-duplication, title/abstract screening, and full-text eligibility (only in English) assessment until the final set of records included 11 studies.

### 2.3. Selection Process

**Figure 1:**
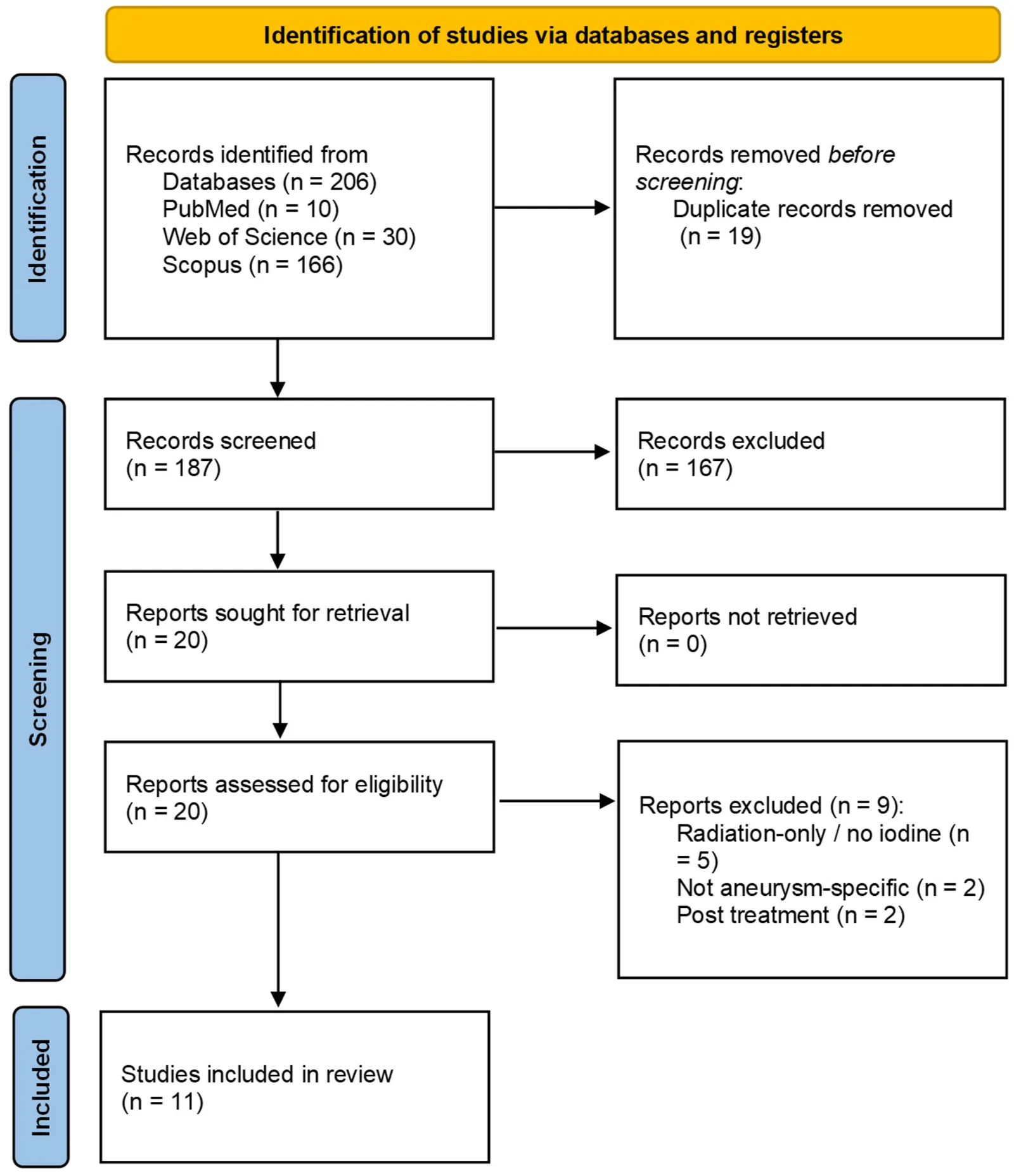
PRISMA flow diagram showing the visual chart of the identified documents, screened and included in this review.

Two reviewers (R.M.L. and R.C.) independently screened titles and abstracts using Rayyan software. Full texts of potentially eligible studies were retrieved and assessed independently by the same two reviewers. Disagreements were resolved by discussion or by consultation with a third senior reviewer (S.D.C.). The selection process is summarized in the PRISMA flow diagram, as depicted in Figure 1. The completed PRISMA 2020 Checklist is provided in the Supplementary Materials (Supplementary File S1).

### 2.4. Data Extraction

Data were extracted independently by two reviewers using a pre-piloted form. Extracted variables included: author, year, country, study design, sample size, unit of analysis, patient population, scanner type, tube voltage, contrast agent name, concentration, volume, injection rate, total iodine load, reconstruction method, reference standard, and outcomes of interest. For studies reporting diagnostic accuracy, true positives, false positives, true negatives, and false negatives were extracted or calculated. Discrepancies were resolved by consensus.

### 2.5. Risk of Bias Assessment

**Figure 2:**
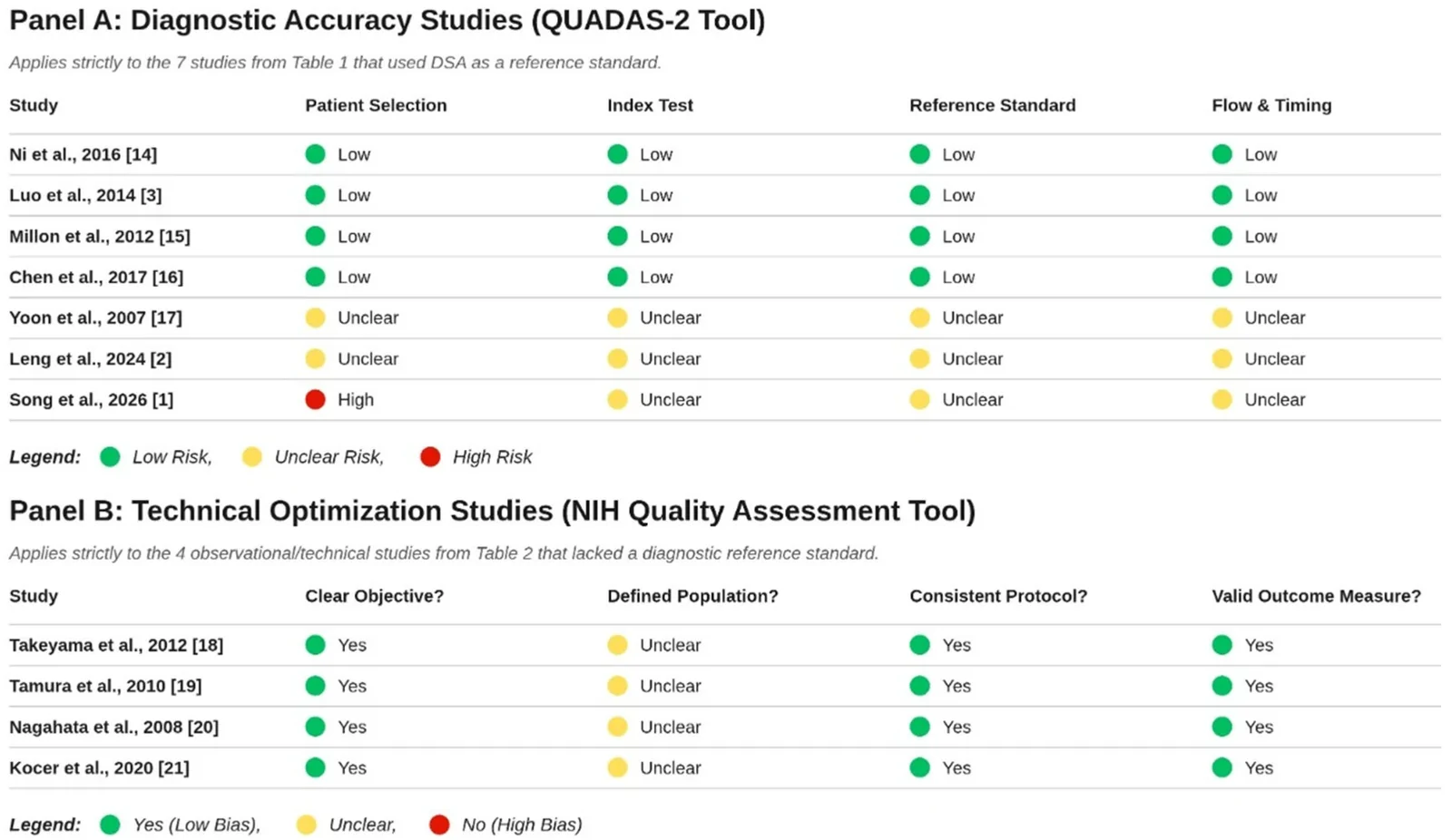
Risk of bias assessment using QUADAS-2 (panel A) and NIH Quality Assessment Tool (Panel B).

Methodological quality was evaluated using a stratified approach based on a primary study design to prevent reporting artifacts (Figure 2). QUADAS-2 was applied for the diagnostic accuracy studies (Panel A) while the NIH Quality Assessment Tool was applied for the technical optimization studies (Panel B).

### 2.6. Data Synthesis

Due to substantial heterogeneity in scanner generations, contrast protocols, patient populations, and outcome measures, a meta-analysis was not performed. A narrative synthesis was conducted, structured by outcome: diagnostic performance, image quality, contrast dose, and radiation dose. Total iodine load was calculated as: volume (mL) × concentration (mgI/mL)/1000 = grams of iodine.

### 2.7. Statistical Analysis

When available, the percentages and confidence intervals for aneurysm detection sensitivity, specificity, predictive value and accuracy, reported in the original articles, were reproduced. Missing values were calculated in MedCalc (MedCalc Software Ltd., Ostend, Belgium, version 23.5) using the raw data reported in each study and the methods implemented by the MedCalc software, which provide the Clopper–Pearson confidence intervals for sensitivity, specificity, accuracy and 0%/100% predictive values, and logit confidence intervals for other predictive values.

## 3. Results

### 3.1. Study Selection and Characteristics

The database search identified 206 records and after duplicate removal, 187 records were screened by title and abstract. Of 20 full-text articles assessed for eligibility, nine were excluded with reasons. Eleven studies were included in the narrative synthesis, as shown in Figure 1. The characteristics of the included studies are summarized in Table 1, comprising a total of 716 patients. Seven studies directly evaluated diagnostic accuracy for aneurysm detection against DSA (Table 1). Four studies evaluated technical image quality parameters without DSA and are considered indirect evidence (Table 2). Sample sizes ranged from 47 to 194 patients. Scanner types included 64-MDCT, 128-MDCT, 320-MDCTCT and dual-layer spectral CT. Tube voltage ranged from 70 to 120 kVp. Contrast volume ranged from 25 mL to 100 mL, with total iodine load ranging from 3.2 g to 30 g.

Beyond the seven studies that directly assessed aneurysm detection, additional studies investigated low-iodine-dose protocols in intracranial CTA but did not report aneurysm-specific diagnostic accuracy. These studies focused on image quality, radiation dose, and technical parameters, and are summarized in Table 2, being identified through the systematic search, but were retained for technical context only and were not used to infer aneurysm diagnostic accuracy.

### 3.2. Diagnostic Performance for Aneurysm Detection

Seven studies compared optimized contrast protocols to standard protocols, with DSA used as the reference standard in all studies. The unit of analysis was patient-level in three studies, aneurysm-level in two studies, and vessel-level in two studies, as detailed in Table 1. In studies using low tube voltage (70–80 kVp) with reduced contrast volume (25 mL to 60 mL), sensitivity ranged from 88.5% [95% CI: 81.6–95.4] to 100% [95% CI: 47.8–100] and specificity ranged from 92.9% [95% CI: 88.6–98.6] to 100% [95% CI: 66.3–100]. Overall accuracy was above 89% across protocols. Most missed aneurysms were <3 mm in size. One study reported eight missed aneurysms <3 mm and two missed aneurysms 3–8 mm in the low-dose group [14]. The evidence for aneurysms <3 mm is inconsistent and limited by small sample sizes and wide confidence intervals [3,16]. Therefore, the diagnostic performance of reduced-dose protocols for very small aneurysms remains uncertain.

### 3.3. Image Quality Metrics

Six studies reported quantitative image quality. Reducing tube voltage from 120 kVp to 70–80 kVp increased arterial attenuation by 35–60% despite a 50% reduction in iodine load [3,6,7,14,15,16]. This is attributable to increased iodine attenuation at low kVp. Iterative reconstruction and deep learning further improved image quality. One study using deep learning with 28 mL at 70 kVp reported significantly higher contrast-to-noise ratio and edge rise slope compared to 40 mL at 100 kVp with filtered back-projection [17]. Subjective 5-point scores for arterial delineation were not significantly different between standard and reduced-dose protocols in four of five studies. The general conclusions were that the acquisition protocols with the reduced doses compare favorably with DSA in terms of diagnostic image quality and diagnostic accuracy. Venous contamination was reduced with lower contrast volumes due to shorter bolus duration.

### 3.4. Contrast Volume and Radiation

Total iodine load was reduced by 50–80% in optimized protocols compared to standard protocols, ranging from 3.2 g to 9 g versus 18 g to 21 g [3,6,7]. Injection rates were maintained between 3.0 and 5.0 mL/s to preserve bolus compactness. Radiation dose was reported in five studies [3,6,14,16,17]. Effective dose ranged from 0.2 ± 0.0 mSv to 1.3 ± 0.1 mSv [16,17]. Low-tube-voltage protocols reduced radiation dose by approximately 23–50% compared to 120 kVp [14,16]. However, dose reduction depends on the complete acquisition protocol, patient size, and iterative reconstruction settings, not on tube voltage alone. No included studies directly assessed the incidence of post-contrast acute kidney injury (PC-AKI). Safety conclusions are therefore limited to technical dose reduction.

### 3.5. Methodological Quality and Risk of Bias Analysis

Methodological quality was evaluated using a stratified approach based on primary study design to prevent reporting artifacts (Figure 2). For the seven clinical diagnostic accuracy trials evaluating CTA against a digital subtraction angiography (DSA) reference standard, the QUADAS-2 framework was applied (Figure 2, Panel A). Four of these trials demonstrated low risk of bias and low applicability concerns across all domains due to prospective consecutive patient recruitment. In contrast, three earlier or highly selected cohorts demonstrated unclear or high risk of bias due to retrospective selection and unquantified indexing windows. For the four technical optimization studies that focused on physical signal validation, flow parameters, or device modeling rather than diagnostic test indices, the NIH Quality Assessment Tool for Observational Cohort and Cross-Sectional Studies was applied (Figure 2, Panel B). All four technical papers demonstrated low risk of bias regarding clear scientific objectives, internal imaging protocols, and physical outcome measures. However, selection bias domains were uniformly categorized as unclear due to a lack of detailed clinical demographic descriptions. This dual-appraisal strategy ensures that technical physics studies are not penalized using criteria designed for pure diagnostic testing.

## 4. Discussion

### 4.1. Main Findings

The current management of interventional neuroradiology and contrast agent administration remains problematic in the context of the lack of numerical guidelines for the application of a principle such as ALADA (As Low As Diagnostically Acceptable) to evaluate and limit exposure [22]. The current guidelines and recommendations do not specify exact values of the injection parameters, and hence, in practice, the injection protocols vary widely in volumes and flow rates. Injection volume, flow rate and pressure should be precisely managed, ensuring homogeneous and reproducible opacification of the vascular region being examined [23]. Iodinated contrast agents are commercially available in different concentrations, ranging from 300 to 400 mgI/mL, while injection volumes commonly range from 20 to 70 mL with flow rates between 3.0 and 7.0 mL/s, adapted to the target vascular region of interest and the acquisition protocol [3,22]. The present systematic review of 11 studies demonstrates that substantial reductions in contrast media volume, often between 30% and 57%, can be safely achieved without sacrificing diagnostic accuracy, sensitivity, or specificity in the evaluation of intracranial aneurysms, as presented in Table 1. Low-tube-voltage techniques (70–80 kVp) combined with advanced iterative reconstruction algorithms allow for contrast reduction down to 25–30 mL while maintaining diagnostic accuracy from 86.3% to 100%. Advanced techniques such as iterative reconstruction and deep learning appear to compensate for the lower signal associated with reduced iodine dose. However, the current evidence base is limited by small sample sizes, heterogeneous protocols, and inconsistent reference standards.

### 4.2. Technical Innovations and Diagnostic Efficiency: Image Quality, Contrast, and Radiation Optimization

**Figure 3:**
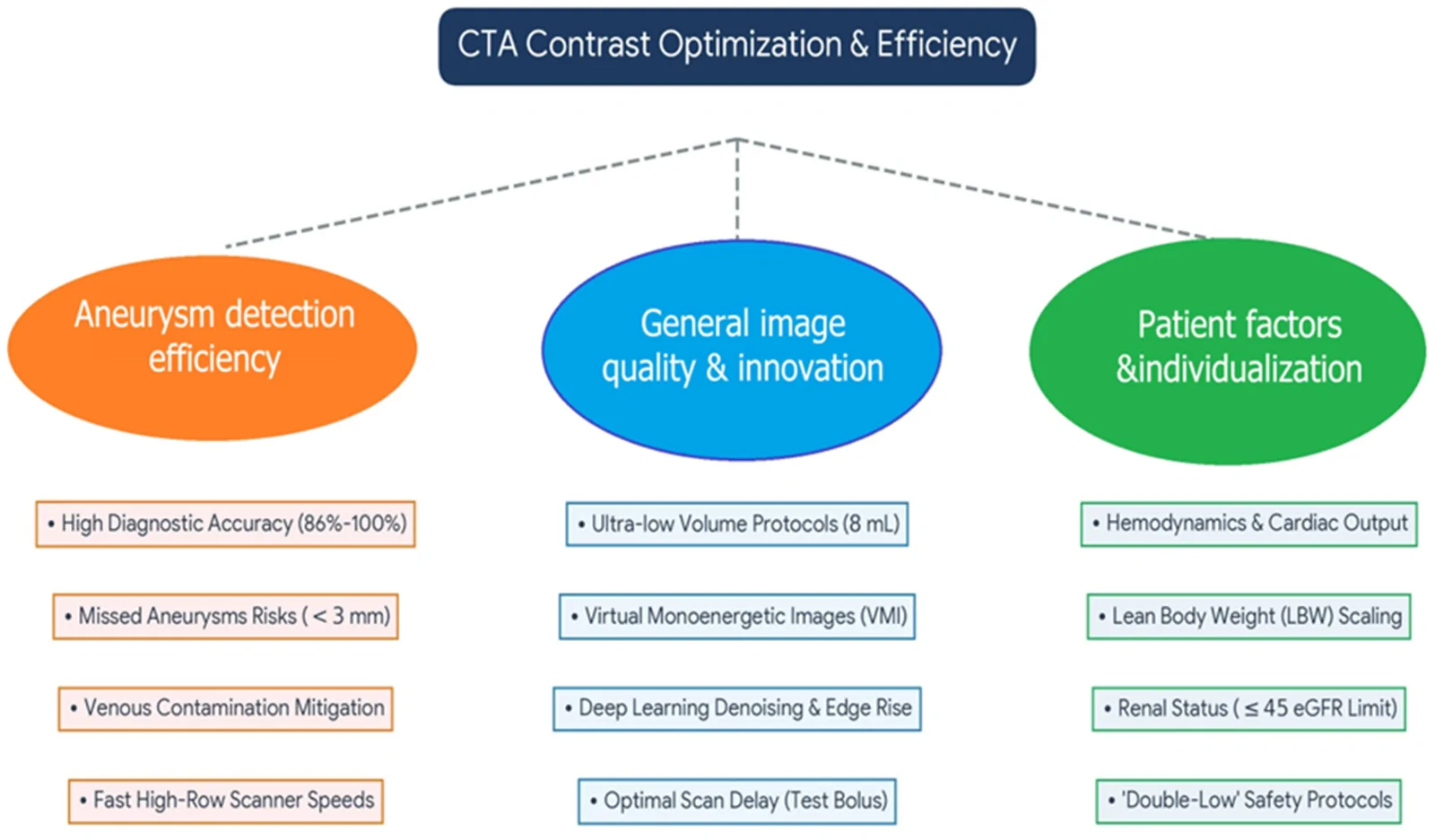
Structural framework of contrast medium optimization and diagnostic efficiency in cerebral computed tomography angiography. The paradigm transitions from empirical “one-size-fits-all” methodologies to individualized imaging based on three symbiotic pillars: preserving diagnostic sensitivity for microaneurysms while mitigating venous contamination; deploying technological advancements such as virtual monoenergetic imaging on spectral detector CT and deep learning denoising to support ultra-low-volume protocols; and individualizing the iodine load based on patient-specific cardiac output, lean body weight and estimated glomerular filtration rate to mitigate contrast-induced nephropathy according to the ALADA.

As illustrated in Figure 3, the optimization of ICA protocols rests upon three interconnected pillars: (1) Diagnostic Efficiency in Aneurysm Detection; (2) Technical Innovations in Image Quality; and (3) Individualized Patient Tailoring.

Lowering tube voltage increases the photoelectric effect of iodine, which significantly boosts vascular attenuation. This counteracts the expected reduction in signal-to-noise ratios when contrast volume is lowered. As shown in the studies summarized in Table 1, Ni et al. [14] reported that CTA with 80 kVp and 30 mL could reduce the effective radiation dose by approximately 73% and the contrast material volume by 50%, without compromising diagnostic yield. However, the signal-to-noise and contrast-to-noise ratios of the internal carotid and middle cerebral arteries decreased due to the fact that the mean image noise increased at the lower voltage. The contrast- and signal-to-noise reduction was mitigated by the simultaneous decrease in the voltage, since the iodinated contrast agent has a higher CT attenuation at low photon energies [16], leading to higher CT attenuation of the vessels despite the lowered iodine dose. A combination of the two reconstruction methods was used by Chen et al. [16], comparing cerebral CTA at 120 kVp with 60 mL contrast material to CTA at 70 kVp with 30 mL contrast material, and applying iterative reconstruction (SAFIRE) on a multidetector-row CTA (Table 1). The study demonstrated that 70 kVp with 30 mL and SAFIRE reconstruction provided diagnostic image quality with 85% lower effective radiation dose.

**Figure 4:**
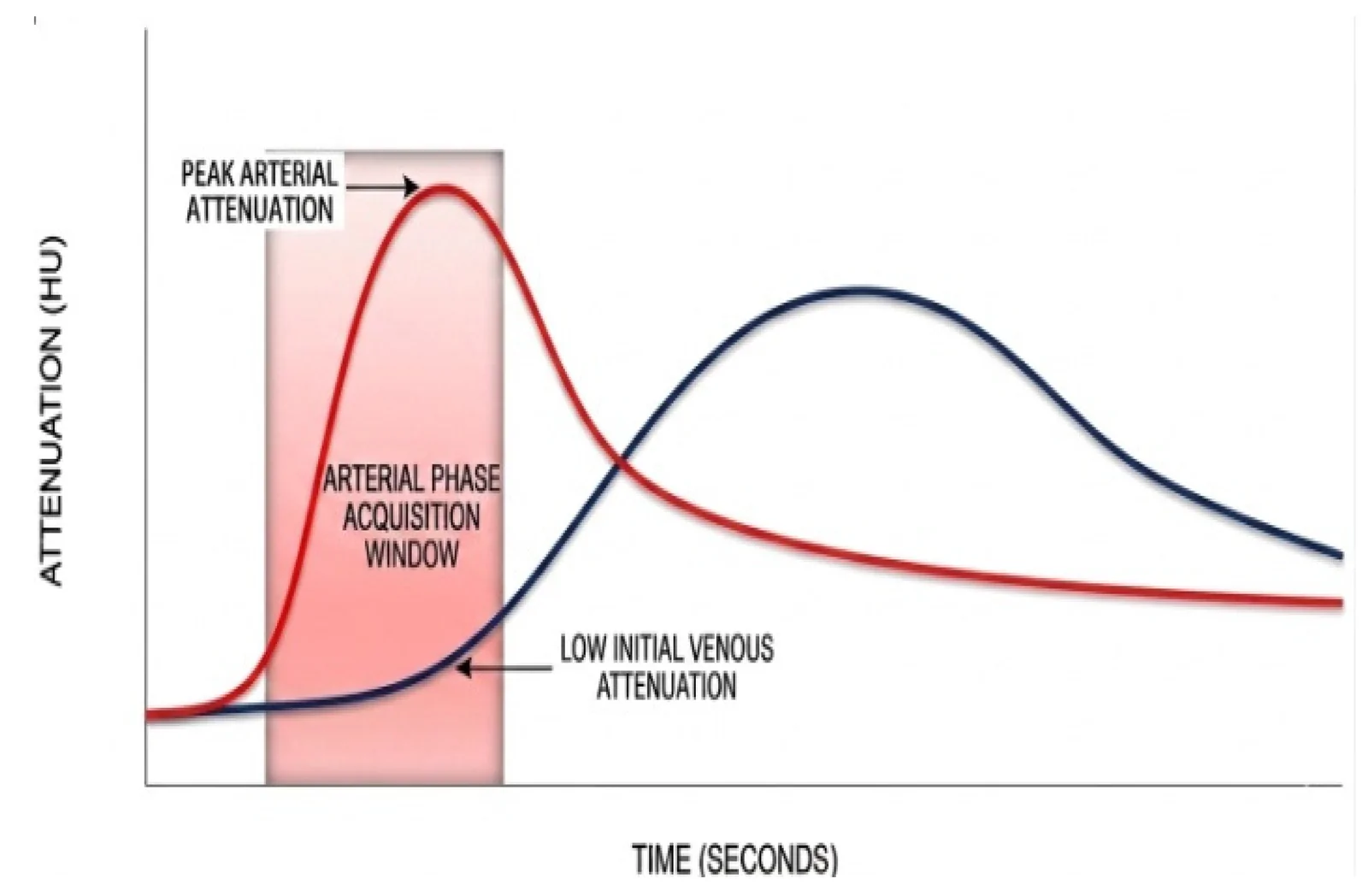
Alignment of the acquisition window with the arterial phase, when arterial attenuation is maximal and venous attenuation is still relatively low (HU—Hounsfield units).

Another study addressing the detection efficiency of intracranial aneurysms when using a low iodine dose [15] evaluated the quality and diagnostic performance of a 25 mL contrast agent using 120 kVp CTA (Table 1). The authors were able to reduce the volume of the contrast medium to 25 mL by optimizing the acquisition timing relative to the arterial and venous phases of the moving bolus, so that the acquisition (with a duration of 3–5 s) has maximum overlap with the arterial phase and minimal overlap with the venous phase (Figure 4). This was also helped by the fact that they used a 64-row detector, as opposed to a single- or four-row detector. Image quality was assessed using a 3-point scale, which classified the image quality based on the degree of venous contamination. Overall, 14 aneurysms (whether ruptured or unruptured) smaller than 4 mm were detected in this study with 100% sensitivity and specificity, while CTA allowed for satisfactory diagnostic and therapeutic management.

On the other hand, Yoon et al. [7] studied three different volumes (100 mL, 80 mL and 60 mL) of contrast material (iohexol, 300 mgI/mL), while maintaining the same voltage (120 kVp on a 16-row MDCT) and similar delivery rates (3–4 mL/s). This approach allows one to isolate the effect of the bolus volume by itself. A qualitative analysis on several 5-point scales assessed intra-arterial contrast, arterial delineation, venous contamination and confidence of diagnosis of vascular lesions. The lowest contrast material volume displayed the lowest mean attenuation, which led to poorer arterial delineation, but less pronounced venous attenuation, which may be related to the shorter delivery time of the bolus (15 s) in this case. Consequently, overall image quality did not differ significantly between the three groups. A larger volume could improve the visualization of distal arterial segments (relative to the Circle of Willis); however, that seems to be of little clinical significance since almost all aneurysms are usually located in the vicinity of the Circle of Willis [24,25]. A rate of 3.5–4.0 mL/s is often “diagnostically acceptable” for larger aneurysms, reducing the risk of contrast extravasation at the injection site [25].

More recently, Song et al. [17] implemented deep learning-based augmented contrast-enhancement and denoising to evaluate the feasibility of a very low iodine dose using a 128-channel scanner. Quantitative analysis focused on the contrast-to-noise ratio, the edge rise distance and the edge rise slope of the signal at the cerebral arteries, while qualitative analysis employed three 5-point scales regarding arterial enhancement, arterial boundary sharpness and image noise. Once again, attenuation values (but also noise) were larger thanks to the reduced voltage in spite of the lowered iodine dose (both volume and concentration). The contrast-to-noise ratio and edge rise slope, as well as the qualitative scores, improved significantly after employing deep learning.

High-row-number detectors allow not only higher spatio-temporal resolution, but also lower scan durations. The lower scan durations, in conjunction with a reduced bolus size, can reduce venous contamination in the final images, while maintaining high arterial attenuation (Figure 4). Too much venous contamination could obscure small and even medium-sized aneurysms in the vicinity of the Circle of Willis. High-row scanners allow for bolus tracking thresholds (typically 100–120 HU) that trigger the scan specifically during the arterial peak before significant return from the cerebral veins [7,26].

The impact of a high detector-row count on the detection efficiency for small aneurysms remains a subject of ongoing debate. While studies in Table 1 suggest that low-tube-voltage scans provide similar or superior accuracy to conventional CTA, other data indicates that sensitivity for ruptured microaneurysms (<3 mm) is significantly lower, ranging from 30% to 78% [27]. Missed aneurysm detections due to low iodine dose occurred usually in the case of aneurysms smaller than 3 mm. The wide confidence intervals and aneurysm-level vs. patient-level analysis in the included studies preclude definitive conclusions. Prospective studies with standardized DSA correlation are needed to confirm performance in this subgroup [7,14,27].

On the other hand, in terms of bolus timing and tracking efficiency, to adhere to the ALADA principle, timing must be flawless to avoid “wasted” contrast. Reducing transit time by minimizing the delay between the start of the injection and the start of the scan can be achieved by reducing the total volume of contrast required to fill the arterial tree [28,29,30,31].

The ultimate application of the ALADA principle is the move toward patient-specific parameters. Factors such as body weight and heart rate significantly influence contrast transit time. By adjusting the injection duration and flow rate to the patient’s specific hemodynamic profile, the systematic review of the current literature suggests that “diagnostic acceptability” can be achieved with significantly lower contrast volumes than was previously standard [30,31,32].

### 4.3. Clinical Implications and Patient-Specific Tailoring

Efficiency in cerebral CTA is achieved only when contrast parameters are tailored to the individual’s physiology. The ultimate application of the ALADA principle is the move toward patient-specific parameters, as follows:

*Hemodynamic Status and Cardiac Output:* The transit time of a contrast bolus is primarily determined by the patient’s cardiac output. In elderly patients or those with congestive heart failure, the bolus arrives later and stays in the arterial system longer. For these patients, a lower flow rate with a longer scan delay or precise bolus tracking is necessary to avoid “missing” the peak opacification of the aneurysm. Clinical consensus recommends using automated bolus tracking or a longer start delay to avoid “missing” the arterial peak, as fixed-delay protocols are highly susceptible to failure in poor hemodynamic conditions [29,30,31,32]. The study by Rau et al. [29] confirms that the arrival of the contrast bolus in the neurocranium is subject to delay and dispersion primarily influenced by cardiac output. Similar research concludes that as cardiac output decreases, the time to peak arterial enhancement significantly increases [31]. The transit time of a contrast bolus is primarily determined by the patient’s cardiac output. For larger aneurysms, a rate of 3.5–4.0 mL/s is often “diagnostically acceptable”, providing sufficient arterial attenuation while balancing the risk of contrast extravasation at the injection site [24,25,28].

*Body Weight and Composition:* Recent research identifies body size as the principal determinant of the volume of distribution for iodinated contrast agents. Fixed-volume protocols cause significant inter-patient variability, leading to excessive enhancement in petite patients and insufficient enhancement in obese patients. Modern protocols recommend adjusting the total iodine load proportionally to the patient’s weight or Body Surface Area. Utilizing lean body weight allows for a significant reduction in contrast volume (often >25%) without compromising diagnostic quality [33,34,35,36].

*Renal Function:* The most significant safety constraint is the patient’s eGFR. For eGFR values between 30 and 45 mL/min/1.73 m^2^, the risk is considered low for intravenous administration but requires individualized consideration. By using 80 kVp imaging, the total contrast volume can be reduced to 30–40 mL, significantly lowering the osmotic load on the kidneys while maintaining diagnostic signal. However, no included studies directly assessed the incidence of post-contrast acute kidney injury (PC-AKI). Safety conclusions are therefore limited to technical dose reduction [14,17,36,37,38,39].

Overall, for patients with good venous access and normal renal function, protocols using 30 mL of 300 mgI/mL at 70–80 kVp with iterative reconstruction may be considered. For patients at higher risk, spectral CT with VMIs may allow further reduction to 8–10 g of total iodine. These strategies should be implemented within institutional protocols and not as a replacement for clinical judgment.

### 4.4. Indirect Technical Evidence

Several studies outside the scope of cerebral aneurysm CTA provide useful technical context but were not used to support primary diagnostic conclusions. These four studies are summarized separately in Table 2. In abdominopelvic CTA, Tamura et al. [19] demonstrated that reducing contrast volume from 100 mL to 50 mL maintained vascular delineation on 16-MDCT. In coronary CTA and general CTA timing, Takeyama et al. [18] evaluated test-bolus methods and scan delay optimization for arterial phase imaging with 40 mL contrast. In early four-slice MDCT work, Nagahata et al. [20] assessed the effect of varying iodine concentration and delivery rate on arteriovenous contrast. In neurointerventional imaging, Kocer et al. [21] described non-diluted contrast at very low flow rates for cone-beam CT evaluation of flow-diverter stents. These studies were identified through the systematic database search but did not evaluate aneurysm detection as an endpoint. They suggest that the underlying physics of low kVp, bolus timing, and contrast optimization are broadly applicable, but direct extrapolation to aneurysm detection is not supported by the current evidence. Future research should test whether these strategies translate to improved aneurysm detection in cerebral CTA.

### 4.5. Limitations

A primary limitation of the current evidence base is the lack of direct, prospective comparison of dose-reduced CTA protocols with DSA in the same patients. Most diagnostic accuracy data for aneurysm detection are derived from retrospective cohorts where DSA was performed prior to CTA, or where surgical findings served as the reference standard. This introduces potential verification bias. Furthermore, the four studies summarized in Table 2 provide only indirect technical evidence and did not evaluate aneurysm detection. Future prospective, paired CTA-DSA studies are needed to definitively validate the diagnostic accuracy and safety of ALADA-guided, low-dose protocols for intracranial aneurysm detection. Second, the number of included studies was small and most were single-center with sample sizes <120 patients. Third, substantial heterogeneity in scanners, protocols, and units of analysis prevented meta-analysis. Fourth, risk of bias was unclear or high in several studies, particularly for the index test domain. Fifth, only a few studies reported outcomes for aneurysms <3 mm, limiting conclusions for this critical subgroup. Sixth, no included study directly assessed clinical endpoints such as PC-AKI.

### 4.6. Novelty Relative to Prior Reviews

While prior reviews have addressed low-kVp CTA, contrast optimization, or spectral CT in general vascular and body imaging, few have focused specifically on intracranial aneurysm detection. The novelty of this review lies in: (1) restricting the analysis to cerebral CTA studies with aneurysm detection endpoints, (2) separating direct diagnostic evidence from indirect technical studies, and (3) integrating patient-specific factors such as cardiac output and lean body weight to guide dose optimization. This provides neurovascular-specific recommendations that are not covered in existing broad reviews of CTA optimization.

## 5. Conclusions

The optimization of iodinated contrast agent (ICA) protocols in multidetector-row computed tomography angiography (CTA) represents a critical paradigm shift from historical “one-size-fits-all” methodologies toward individualized, patient-centric imaging. Guided by the ALADA (As Low As Diagnostically Acceptable) principle, recent clinical evidence demonstrates that reduced-contrast, low-kVp CTA protocols combined with iterative reconstruction and deep learning techniques may preserve diagnostic performance for intracranial aneurysm detection while lowering iodine exposure by 30–57% and radiation dose by up to 74%. Evidence from diagnostic studies supports sensitivities of 86.3–100% and specificities of 92.9–100% with contrast volumes as low as 25–30 mL, particularly when bolus tracking at 100–120 HU and high-row detectors are used to minimize venous contamination. Patient-specific tailoring using lean body weight and cardiac output may further optimize dosing and reduce renal risk in vulnerable populations. For vulnerable cohorts presenting with pre-existing renal insufficiency, “double-low” (low-radiation, low-contrast) strategies minimize the osmotic load on the kidneys, offering a proactive, highly effective approach to mitigating contrast-induced nephropathy. However, current evidence is limited by retrospective designs, heterogeneity, and lack of standardized reporting of PC-AKI and radiation dose. Studies providing indirect technical context should not be extrapolated to aneurysm detection without direct validation. The lack of direct prospective CTA-to-DSA comparison in most studies remains a key limitation and warrants future validation. Prospective, multi-center, aneurysm-specific studies with standardized protocols, DSA validation, and safety outcomes are needed to confirm these findings and to establish evidence-based, ALADA-guided guidelines for contrast optimization in cerebral CTA.

## Figures and Tables

**Table 1:** Summary of included studies evaluating contrast optimization in cerebral CTA for intracranial aneurysms.

Author, Year/Ref	Country	Study Design/Unit of Analysis	Population	CT Scanner and Tube Voltage	Contrast Protocol (Iodine Volume, Injection Speed)	Key Finding	Outcome Measured
Ni et al., 2016 [14]	China	Retrospective/Aneurysm-level	102	64-detector CT 80 kVp/ 120 kVp	30 mL respectively 60 mL, speed 4 mL/s + saline flush	Low voltage improved CNR	Image quality, aneurysm detection
Luo et al., 2014 [3]	China	Prospective/Patient-level	120	320-detector CT 120 kVp/ 100 kVp/ 80 kVp	70 mL respectively 30 mL, speed 5 mL/s, bolus tracking	Reduced contrast volume maintained diagnostic accuracy	Contrast dose, SNR improved
Millon et al., 2012 [15]	France	Retrospective/Aneurysm-level	73	64-detector CT 120 kVp	25 mL, 5 mL/s	Test-bolus technique optimized timing	Ruptured/unruptured aneurysms <4 mm detected with 100% sensitivity and specificity
Chen et al., 2017 [16]	Taiwan	Prospective/Patient-level	100	128-detector CT 120 kVp 70 kVp	30 mL respectively 60 mL, speed 4 mL/s + dual-energy	DE-CTA improved vessel contrast	Radiation dose, diagnostic performance
Yoon et al., 2007 [7]	Korea	Retrospective/Vessel-level	194	16-detector CT 120 kVp	100 mL, 80 mL and respectively 60 mL, speed 3.5 mL/s	Higher volume needed for older scanners	Image quality score
Leng et al., 2024 [6]	USA	Prospective/Patient-level	80	256-detector CT 120 kVp	40 mL speed 4 mL/s, P3T injector	Ultra-low contrast protocol feasible	Significantly higher SNR and CNR for low volume
Song et al., 2026 [17]	China	Retrospective/Vessel-level	47	320-detector CT 100 kVp 70 kVp	40 mL respectively 28 mL speed 2.5 mL/s + AI reconstruction	AI denoising allowed 30% contrast reduction	Low voltage CT and ultra-low iodine load achieved higher arterial attenuation, superior CNR

Abbreviations: CNR = contrast-to-noise ratio, SNR = signal-to-noise ratio, DE-CTA = dual-energy CTA, and kVp = kilovoltage peak.

**Table 2:** Summary of studies evaluating low-iodine-dose protocols in intracranial CTA without aneurysm-specific outcomes.

Author, Year/Ref.	Country	Population	Study Aim	Iodine Protocol/Tube Voltage	Main Outcome
Takeyama et al., 2012 [18]	Japan	NR	Determine optimal scan delay with test bolus	40 mL (370 mgI/mL, 4 mL/s)/120 kVp	Optimal delay maximized arterial enhancement.
Tamura et al., 2010 [19]	Japan	NR	Compare contrast volume with/without saline flush	100 mL vs. 50 mL (300 mgI/mL, 3 mL/s)/120 kVp	No significant difference in vascular delineation.
Nagahata et al., 2008 [20]	Japan	NR	Evaluate effect of contrast parameters	100 mL (300 mgI/mL) vs. 80 mL (370 mgI/mL)/140 kVp	Higher concentration increased arteriovenous contrast.
Kocer et al., 2020 [21]	Turkey	NR	Evaluate flow-diverter stents with very low rate	96 mL (30 mgI/mL) vs. 9.6 mL (300 mgI/mL, 0.4 mL/s)/80 kVp	Non-diluted cone-beam CT suitable for stent evaluation.

NR = Not Reported.

## Data Availability

No new data were created or analyzed in this study. Data sharing is not applicable to this article.

## Supplementary Materials

The following supporting information can be downloaded at: https://www.mdpi.com/article/10.3390/diagnostics16162666/s1, File S1: PRISMA checklist.

## Abbreviations

The following abbreviations are used in this manuscript:

ALADA	As Low As Diagnostically Acceptable
BMI	Body Mass Index
BSA	Body Surface Area
CBCT	Cone-Beam Computed Tomography
CI	Confidence Interval
CIN	Contrast-Induced Nephropathy
CNR	Contrast-to-Noise Ratio
CO	Cardiac Output
CTA	Computed Tomography Angiography
DSA	Digital Subtraction Angiography
eGFR	Estimated Glomerular Filtration Rate
FBP	Filtered Back-Projection
FN	False Negative
FP	False Positive
HU	Hounsfield Unit
ICA	Iodinated Contrast Agent(s)
kV	Kilovolt
kVp	Kilovoltage peak
LBW	Lean Body Weight
mgI/mL	Milligrams of iodine per milliliter
MDCT	Multidetector Computed Tomography
MeSH	Medical Subject Headings
mL	Milliliter
mL/s	Milliliters per second
N/A	(NAv) not applicable (not available)
NPV	Negative Predictive Value
PCI	Polychromatic Image
PPV	Positive Predictive Value
PRISMA	Preferred Reporting Items for Systematic Reviews and Meta-Analyses
ROI	Region of Interest
SAFIRE	Sinogram-Affirmed Iterative Reconstruction
SAH	Subarachnoid Hemorrhage
SDCT	Spectral Detector Computed Tomography
SNR	Signal-to-Noise Ratio
TN	True Negative
TP	True Positive
VMI	Virtual Monoenergetic Image
